# Heterogeneity of immune infiltration and immunotherapy in colorectal cancer

**DOI:** 10.1093/gastro/goad079

**Published:** 2024-02-16

**Authors:** Yichen Li, Jun Hu, Min Zhi

**Affiliations:** School of Medicine, Jiaying University, Meizhou, Guangdong, P. R. China; Zhongshan School of Medicine, Sun Yat-sen University, Guangzhou, Guangdong, P. R. China; Guangdong Provincial Key Laboratory of Colorectal and Pelvic Floor Disease, The Sixth Affiliated Hospital, Sun Yat-sen University, Guangzhou, Guangdong, P. R. China; Department of Gastroenterology, The Sixth Affiliated Hospital, Sun Yat-sen University, Guangzhou, Guangdong, P. R. China; Guangdong Provincial Key Laboratory of Colorectal and Pelvic Floor Disease, The Sixth Affiliated Hospital, Sun Yat-sen University, Guangzhou, Guangdong, P. R. China; Department of Gastroenterology, The Sixth Affiliated Hospital, Sun Yat-sen University, Guangzhou, Guangdong, P. R. China

Wu *et al*. [1] recently published a study on potential prognostic and therapeutic values of immune cell infiltration (ICI) subtypes in colorectal cancer (CRC). In this study, they creatively designed a new approach combining the CIBERSORT and ESTIMATE methods to assess the differences in the intra-tumor immune infiltration landscape of CRC samples and its association with prognosis. They found that tumors with a high ICI score had a better prognosis, which was characterized by increased tumor mutational burden (TMB), increased expression of genes involved in immune checkpoints, chemokines, and the interleukin-17 (IL-17) signaling pathway. These results may help predict the prognosis of CRC patients receiving immune checkpoint therapy (ICT) and identify new potential immunotherapy targets for CRC.

Immunotherapy has made great strides in the treatment of CRC, whereas ICT is effective in deficient mismatch repair (dMMR)/microsatellite instability–high (MSI-H) subtypes that comprise only ∼10% of these subtypes and is virtually unresponsive in the proficient mismatch repair (pMMR)/microsatellite stable (MSS) subtypes that make up the majority [2, 3]. Even in CRC with dMMR/MSI-H, the response rate is ∼40% [4]. The tumor microenvironment (TME) is thought to be critical for ICT response, so profiling the immune heterogeneity of the TME may help to screen the population for benefit from immunotherapy. Differences in immune infiltration also lead to differences in ICT efficacy in dMMR/MSI-H subtypes. A recent study found that ICT is more effective in consensus molecular subtypes type 1 dMMR/MSI-H gastrointestinal tumors [5]. Gallois *et al*. [6] proposed predictive subtyping of susceptibility and resistance to ICT for the treatment of MSI-H metastatic CRC. Biomarkers for ICT efficacy in dMMR/MSI-H CRC are continuing to be investigated. Although ICT alone has been shown to be ineffective in pMMR/MSS CRC, it is not clear whether a small proportion of ICT-sensitive immuno-activated subtypes exist in these low-TMB tumors. The ICI score proposed in this study may help to screen for the immune activated subtypes of pMMR/MSS. Researchers are investigating the efficacy of various immunotherapies (chemotherapy, tyrosine kinase inhibitors, etc.) or their combinations in pMMR/MSS CRC and results from these clinical trials and parallel marker studies will help to answer the following questions: Is there a small subpopulation of pMMR/MSS with similar immune infiltration to dMMR/MSI-H? What are the ICT efficacy and prognosis of this subgroup? Can combination therapy make ICT successful in pMMR/MSS?

How TME heterogeneity arises and is remodeled by immunity is poorly understood. A recent study by the same group described immune and stromal cell remodeling in dMMR/MSI-H CRC during tumor complete response, providing valuable insights into the mechanism of immunotherapy [7]. Similar studies have been conducted in head and neck cancer, non-small-cell lung cancer, and triple-negative breast cancer [8–10]. For example, a tissue resident memory subpopulation of CD8 T cells, Treg cells, and myeloid clusters including dendritic cells and macrophages seems to affect the efficacy of ICT. For the pMMR/MSS subtype, which accounts for the majority of CRC, studies on antitumor mechanisms and efficacy markers for specific ICT and ICT combination regimens are urgently needed to better expand immunotherapy indications and to identify novel targets. At present, single-cell-based techniques including mass spectrometry, flow cytometry, and single-cell sequencing offer enhanced insights into the TME. However, their clinical application is constrained by cost and sample quality prerequisites. Finding the best TME indicators for use in clinical practice will require the integration of multi-omics assays and analysis pipelines in future research to explore TME heterogeneity associated with specific biological processes.

Along this line, future research should engage in the following areas: expanding the sample size of the study cohort, stratifying pMMR/MSS and dMMR/MSI-H subtypes, correlating treatment response with immune infiltration scores, improving immune infiltration scores by integrating single-cell dimensional TME features, and developing the immune infiltration score into a clinically accessible panel. It is becoming increasingly clear that the treatment efficacy of ICT in early dMMR/MSI-H CRC is better than in metastatic dMMR/MSI-H CRC. Whether this is due to the different TME patterns or different combinations of ICI scores with metastatic staging is worthy of further investigation.
